# Toward 100 Mega-Frames per Second: Design of an Ultimate Ultra-High-Speed Image Sensor

**DOI:** 10.3390/s100100016

**Published:** 2009-12-24

**Authors:** Vu Truong Son Dao, Takeharu Goji Etoh, Masatoshi Tanaka, Hoang Dung Nguyen, Vo Le Cuong, Kohsei Takehara, Toshiro Akino, Kenji Nishi, Hitoshi Aoki, Junichi Nakai

**Affiliations:** 1 Graduate School of Science and Engineering, Kinki University, Higashi-Osaka, Osaka 577-8502, Japan; E-Mails: best2010@civileng.kindai.ac.jp (T.G.E.); mtanaka@civileng.kindai.ac.jp (M.T.); hdnguyen@civileng.kindai.ac.jp (N.H.D.); vlcuong@civileng.kindai.ac.jp (V.L.C.); takehara@civileng.kindai.ac.jp (K.T.); 2 School of Biology-Oriented Science and Technology, Kinki University, Wakayama 649-6493, Japan; E-Mail: akino@info.waka.kindai.ac.jp; 3 Kinki University Technical College, Kumano, Mie 519-4935, Japan; E-Mail: nishi@ktc.ac.jp; 4 Sharp Corporation, Fukuyama, Hiroshima 721-8522, Japan; E-Mails: aoki.hitoshi@sharp.co.jp (H.O.); nakai.junichi@sharp.co.jp (J.N.)

**Keywords:** backside illumination, CCD, high speed, high sensitivity, image sensor, ISIS

## Abstract

Our experience in the design of an ultra-high speed image sensor targeting the theoretical maximum frame rate is summarized. The imager is the backside illuminated *in situ* storage image sensor (BSI ISIS). It is confirmed that the critical factor limiting the highest frame rate is the signal electron transit time from the generation layer at the back side of each pixel to the input gate to the *in situ* storage area on the front side. The theoretical maximum frame rate is estimated at 100 Mega-frames per second (Mfps) by transient simulation study. The sensor has a spatial resolution of 140,800 pixels with 126 linear storage elements installed in each pixel. The very high sensitivity is ensured by application of backside illumination technology and cooling. The ultra-high frame rate is achieved by the *in situ* storage image sensor (ISIS) structure on the front side. In this paper, we summarize technologies developed to achieve the theoretical maximum frame rate, including: (1) a special p-well design by triple injections to generate a smooth electric field backside towards the collection gate on the front side, resulting in much shorter electron transit time; (2) design technique to reduce RC delay by employing an extra metal layer exclusively to electrodes responsible for ultra-high speed image capturing; (3) a CCD specific complementary on-chip inductance minimization technique with a couple of stacked differential bus lines.

## 1. Introduction

### 1.1. Evolution of the CCD-ISIS

Image sensors for high-speed image capturing may be categorized as follows:

1. Parallel/Partial Readout Imaging Scheme (PPR):
  This is the most common technique to increase the frame rate simply by reading out image signals in parallel through many readout taps, and/or partially only from a selected area in the imaging area.
2. *In situ* Storage Imaging Scheme (ISIS):
  A number of storage elements are placed within or nearby each pixel to store a number of consecutively captured images. The ISIS camera operates at an ultra-high frame rate with a frame interval equal to the time required for signal electrons generated in photo-diodes move to the nearby storage elements [1–6].

The PPR cameras can provide higher spatial resolution when operating at reasonably high frame rates. Currently, advanced PPR cameras achieve several thousand frames per second (fps) for more than 1,000,000 pixels [7]. However, the pixel count reduces when the cameras operate at a higher frame rate. For example, a camera of this type can capture images at more than 1,000,000 fps, but the pixel count reduces to less than 64 × 16. The number of continuous frames is practically unlimited, since the image signals are continuously read out from the image sensor and stored in a large size buffer memory device outside the sensor.

An ISIS camera has achieved 1,000,000 fps (1 Mfps) [2,3]. The frame rate is not limited by the pixel count. However, the number of continuous frames is severely limited by the number of storage elements installable in a small area nearby each pixel. The number of *in situ* memory elements should be more than 100, considering that replay at 10 fps makes moving images look smooth and replay for more than 10 seconds at 10 fps enables scientists and engineers fully recognize dynamic events. Image signals stored in the *in situ* storage are read out slowly from the sensor after cease of each continuous image acquisition phase.

Most of the *in situ* storage image sensors are CCD imagers [1–6], whereas some are based on the CMOS imager technology [8]. Kosonocky *et al.* proposed and fabricated a CCD ISIS, and proved the validity of the in-pixel storage scheme [1]. Each pixel is equipped with an SPS (Series-Parallel-Series) CCD register, which transfer signal charges in two directions in a pixel, *i.e.*, horizontally, vertically and then horizontally again during image capturing. The sensor is capable of recording 30 consecutive images at 833,000 fps.

In 2001, Etoh *et al.* developed a CCD image sensor in which a slanted linear CCD register is installed in each pixel to achieve a simplest one-direction charge transfer. The camera records 103 consecutive 81,120-pixel images at a speed of 1 Mfps [2,3]. Ohtake *et al.*, supervised by Etoh, developed the color version with 300,000 pixels capable of recording 144 consecutive frames while maintaining the maximum frame rate at 1 Mfps (ISIS-V4) [4]. These cameras have been extensively applied to high-speed fluid dynamics [9].

In 2007, Lazovsky *et al.* proposed a CCD structure that can record only 16 consecutive images of 64 × 64 pixels at the speed of 100 Mfps [6]. Due to inexistence of continuous overwriting mechanism, low fill factor and low quantum efficiency, the sensor has limited practical uses.

Some technologies can capture consecutive images at much higher frame rates. Shiraga *et al.* achieved a frame interval down to several picoseconds by using electronic streak combined with a linear slanted imaging scheme, which is similar to the slanted linear CCD storage [10]. By implementing an innovative holographic imaging technology, Kubota *et al.* successfully captured three-dimensional moving images of flying light of a frame interval in the order of femtoseconds [11]. Goda *et al.* developed a technology that can take images at a frame rate of 6.1 millions fps with a single photo-detector [12]. Even conventional multi-framing cameras operate at frame rates higher than 1 Mfps.

However, the ISIS cameras [2–5] provide much better image quality, higher spatial resolution and wider applicability with the simple and compact structure. ISIS cameras have been tested for applicability in bio- and nano-science and engineering. For the tests, the cameras were mounted on microscopes and TEMs. However, most of the attempts failed. The sensitivity of the image sensors is insufficient; the incident light is too weak due to a very short frame interval and a very high magnification rate.

Therefore, the authors started development of new image sensors that satisfy the two requirements: ultra-high speed and ultra-high sensitivity. The sensor is a backside illuminated ISIS (BSI-ISIS) [5]. The very high frame rate is achieved by employment of the ISIS structure on the front side; the very high sensitivity achieved by a combined use of three existing technologies: backside illumination, cooling and CCM, *i.e.*, the multi-step impact ionization [14]. CCD image sensors equipped with CCM are referred as EM-CCD and widely used for fluorescent imaging in bio-science.

The test BSI-ISIS has been fabricated and evaluated (ISIS-V12) [5,13]. The pixel count is 200,000; the full well capacity is 10,000e-. The maximum frame rate is 250,000 fps and 1 Mfps, respectively for full well capacity and reduced charge handling capacity. The sensitivity is very high: images with a 4.9e- signal level in the 7.7e- noise floor was observed. It is noteworthy that the BSI-ISIS which utilizes the CCM can detect signals with levels lower than the noise floor.

The authors thus have started designing in parallel the following two new BSI-ISISes:

1. A BSI-ISIS with the maximum frame rate achievable by a current standard CCD technology.
2. A BSI-ISIS with the theoretical maximum frame rate.

The first one is currently in the final stage of fabrication. The frame rate and the sensitivity are expected to be 10 Mfps and sub-ten-photon per pixel, respectively (ISIS-MV12). The design has been briefly explained elsewhere [5]. The theoretical maximum frame rate for the BSI-ISIS is estimated about 100 Mfps, as explained later. The design has been completed. It was named ISIS-100M after its targeted frame rate. The design requires three metal layers. In addition, to achieve 100 Mfps, two metal layers are necessary on the carrier substrate to send driving voltages through stacked differential wires.

Development of the CCD-ISIS family is summarized in Table 1.

Current standard CMOS processes utilize four or more metal layers. CCD can be fabricated by advanced CMOS processes with the fine design rule with slight modification [15]. Therefore, the authors started the modification of the ISIS-100M to fit an advanced CMOS process. The CMOS-based ISIS adds another function, PPR, the parallel and partial readout, to the ultra-high speed and very high sensitivity of the CCD-ISIS. The sensor is named as RA-ISIS, after its function: the random-access and the *in situ* storage image signal. Reducing the chip size is another way to increase the frame rate. A fine CMOS process effectively reduces the pixel size of the CCD-ISIS. The RA-ISIS will realize the ultimate ultra-high-speed image sensor.

### 1.2. The Design Experience of ISIS-100M

This paper summarizes the work on design of the ISIS-100M. After describing the general structure, we present technologies to accelerate transfer speed of signal electrons from the backside to the front side, which include designs of the dedicated special wafer, the special p-well design and preliminary evaluation of effectiveness of a microlens array. We also introduce technologies to deliver driving voltage with less attenuation. These include wiring design methods of the bus lines by an original evaluation method of the resistive-capacitive delay (RC-delay) and with an innovative CCD-specific complementary bus line structure. Then, the design modification of the ISIS-100M for fabrication with a CMOS process is briefly described.

## 2. General Description of the ISIS-100M

### 2.1. Plane and Cross Section Structure

**Figure 1.**
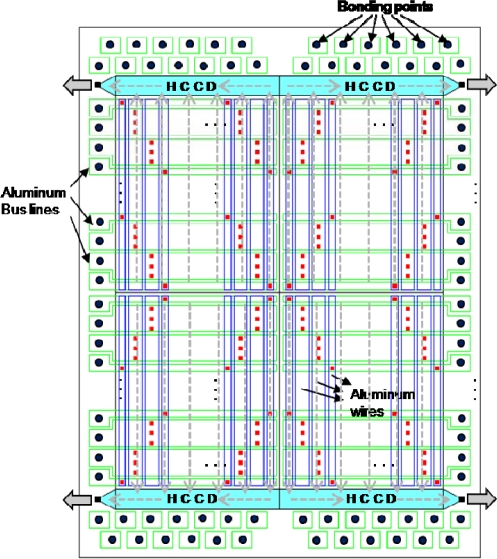
Architecture of the ISIS-100M chip.

**Figure 2.**
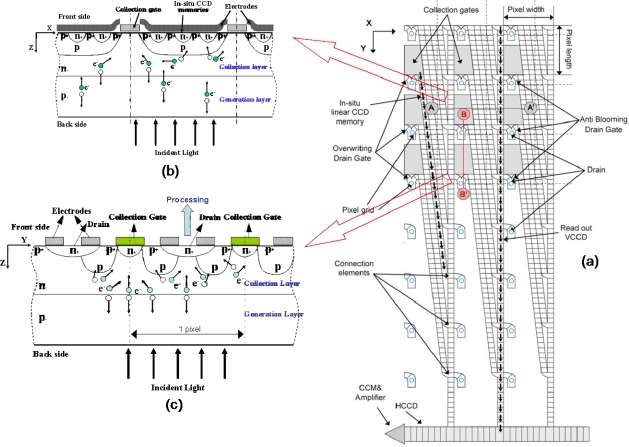
The ISIS-100M structure (a) Front-side ISIS structure (b) Cross-section structure along A-A′ (c) Cross section structure along B-B’.

The ISIS-100M has four symmetrical blocks, as shown in Figure 1. Each quadrant contains one photoreceptive area, one horizontal readout CCD channel, one floating diffusion amplifier and one read-out tap. Each pixel element is equipped with a collection gate, a linear storage area and a drain attached at the end of each CCD storage channel. The plane and cross-section structures are shown in Figure 2.

When an incident photon generates an electron-hole pair in the thick p- generation layer, the photo-electron then travels vertically from the generation layer to the n- collection layer, horizontally in the n- collection layer and again, vertically to the n+ buried channel under collection gate to make a signal charge packet. The charge packet is then transferred to the *in situ* CCD storage elements.

The captured image signals are simultaneously stored in the linear CCD storage area stretching vertically downward from the collection gate. This feature enables the ultimate ultra-high-speed continuous image capturing.

At the end of the linear memory CCD of each pixel, a drain is installed. Image signals from the collection gate are transferred on the CCD and finally reach the drain, from which they are continuously drained out of the sensor. With this overwriting recording operation, the latest image signals are always stored in the linear memory CCD. Once occurrence of a target event is detected, the overwriting operation stops and the image signals stored inside the sensor are slowly read out.

### 2.2. Basic Performance

As shown in Table 1, the target frame of ISIS-100M is 100 Mfps and the pixel count is 140,800. The number of the consecutive frames is 126 and the pixel size is 50.4 × 50.4 μm^2^, respectively. Very high sensitivity is expected with the BSI-CCD structure, cooling and the large pixel size. The expected performance indices are estimated by simulations and shown in Table 2.

## 3. Technologies to Achieve Ultra-high Frame Rate

To achieve the ultra-high frame rate, the following technologies are used, some of which are our original innovative technologies:

1. Two-phase-transfer CCD: Although the two-phase transfer is a common CCD technology, it increases the frame rate when combined with differential driving voltage transfer. However, employment of the two-phase transfer functions to increase the frame rate by the combination with the complementary internal bus lines explained later. The contact structures between the polysilicon and metal layers are simplified, which makes it easy to employ multi-metal layers to reduce parasitic impedance in transfer of the driving voltages.
2. The wafer with double-epi layers: To form the cross-section structure shown in Figures 2 (b) and (c), a dedicated wafer with n- and p-double epi-layers with gradated concentration profiles is used as the starting material [16].
3. Three-layer p-well design: An innovative design with three p-well layers is introduced to generate a smoothly changing potential gradient toward the collection gate and to protect the storage CCD channels from migration of generated photoelectrons. The p-well of each pixel has two holes: a large one at the collection gate is to introduce photoelectrons to the storage CCD channels on the front side; a small one at the drain to collect five percent of the generated photo-electrons to monitor in real time a sudden change in the average brightness, which serves as an index for occurrence of a target event.
4. Curved CCD design: Since the very early development stage of the CCD-ISIS group, a curved design has been introduced to transfer photoelectrons smoothly and swiftly [17].
5. Microlens array: In design of common imagers, microlens arrays are mainly used to increase the nominal fill factor. For BSI-ISIS, they contribute to pixel separation for oblique incident light to increase the frame rate.
6. Wiring layout to minimize RC delay in driving voltage transfer: Attenuation of driving voltages of the ISIS-V12 is rather high, which limited the maximum frame rate for the full well capacity at 250,000 fps. We developed a simple yet accurate evaluation method to estimate the attenuation of the sensor without time-consuming full-scale circuit simulation [18].
7. Complementary global bus-lines: An innovative, CCD-specific design is introduced to reduce the inductance of the two-phase bus lines. This is required to allow the high frequency current flows needed to drive the ISIS-100M at 100 Mfps.

Among these technologies, the wafer with double-epi layers [16], the curved CCD design method [17] and an optimization method of wiring for driving voltage transfer to minimize RC delay [18] have been explained elsewhere. The present paper only describes the effectiveness of the three-layer p-well design, the microlens array and the CCD-specific complementary bus-line design in increasing the frame rate.

## 4. Acceleration of Photo-Electrons Transport from the Backside to Collection Gate

### 4.1. Special P-well Layer Design

A problem specific to the ISIS is direct intrusion of incident light from the backside to the storage area on the front side, where previous signal electrons have been stored. Another one is migration of photo-electrons to the storage area. These problems become serious especially during shutter closing when signal electrons are kept in the storage area without being transferred. It takes several milliseconds, which is much longer than the frame interval.

The thickness of the epi-layers is 30 micrometers, which is sufficient to protect the storage CCD on the front side from direct intrusion of incident light with wavelengths less than 650 nm to the backside. The n^-^ and p^-^ epi-layers are respectively 10 and 20 micrometers thick.

**Figure 3.**
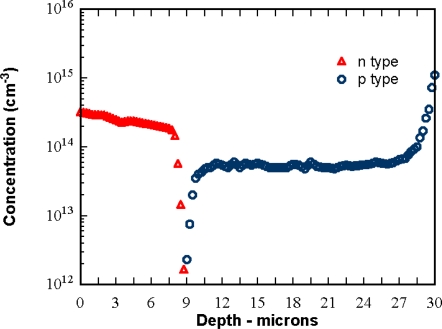
A concentration profile of the gradated double-epi wafer: measurement result by Spreading Resistance (SR) method.

To reduce the electron transit time from the backside to the front side and to create p-well in the n- layer, a special wafer with double-epi layers with gradated concentration profile was developed. Figure 3 shows an example of the concentration profile. To make the epi-layers fully depleted, a negative bias voltage is applied to the backside of the sensor.

In the n-epi layer, we created a p-well with a special potential profile by using three masks. The purposes are four-fold:

1. To confine photo-electrons generated in the generation layer at the backside within the pixel area.
2. To make a p-well protect the storage area from migration of signal electrons.
3. To accelerate their horizontal transfer speed.
4. To smoothly introduce them to the collection gate and to the input gate under the p-well.

**Figure 4.**
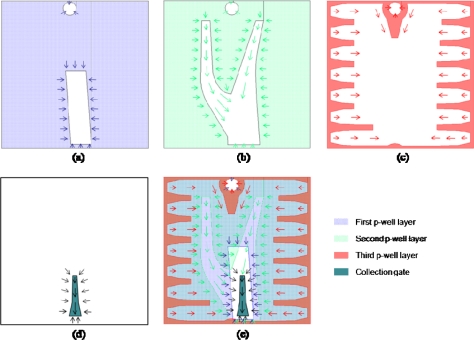
Designs of p-well and collection gate. (a), (b), (c): the first, second and third design masks of the p-well; (d) collection gate; and (e) superimposed p-well design. Arrows indicate transfer direction of photoelectrons.

The designs of the three p-well layers are shown in Figure 4, each with the same doping concentration and the shape optimized for each function:

1. The first layer covers most of the pixel area except the collection gate, which creates a horizontal barrier.
2. The second layer also covers most of the pixel area and also serves as a horizontal barrier. Besides, there are two trunk-like large holes stretching toward the top left and top right corner of the pixel, respectively. The holes were designed to have a widening exponential shape toward the collection gate to generate a potential gradient vertically downward the collection gate. The bifurcation of this layer design is introduced to separate the electron path to the collection gate from the path to the drain through the small hole for a video trigger.
3. The last p-well layer consists of a finger-like structure around the pixel perimeter to define pixel boundaries. It also has one trapezoid stretching downward from the small hole to separate the electron paths.

**Figure 5.**
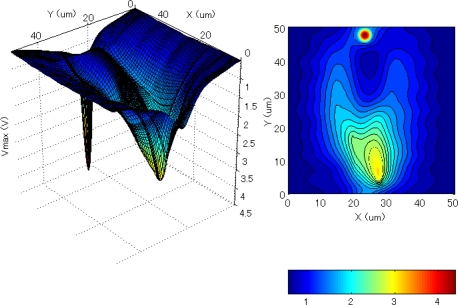
Potential profile in the deep part of a fundamental pixel.

**Figure 6.**
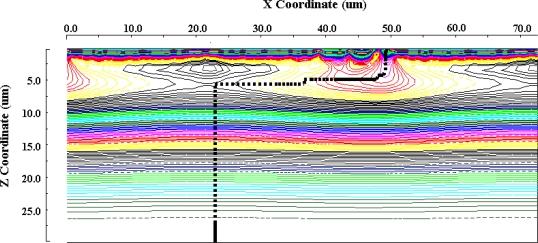
Cross section profile and an electron path from the backside of the sensor to the collection gate.

In Figure 4, a collection gate is also depicted beneath the large rectangular hole. The electrons are finally transferred from the collection gate to the input gate, which is the first element of the storage CCD placed under the collection gate (not drawn). Figures 5 and 6 show the potential profile and the cross section profile created by the special p-well design. The potential changes smoothly toward the collection gate and to the drain.

### 4.2. Electron Transit Time and Efficiency of Micro-Lens Array

**Figure 7.**
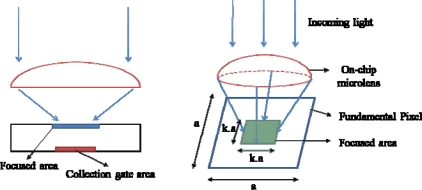
Function of on-chip micro-lens array concept (k is light collection rate).

To evaluate the maximum frame rate, we have to assume the operation scheme of the sensor. The frame interval is 10 ns for 100 Mfps. If the integration time of signal electrons is 5 ns and electrons generated during another 5 ns are drained by the electronic shutter function of the input gate, the signal electrons need to be collected in the collection gate within 5 ns. In addition to this operation scheme, the following conditions are assumed for preliminary evaluation:

1. Ten thousands photoelectrons are generated at once at and distributed uniformly over the back surface of each pixel.
2. A microlens on the pixel collects and uniformly distributes the electrons in the central area of the back surface: the area is 50% or 20% of the back surface as depicted in Figure 7.

**Figure 8.**
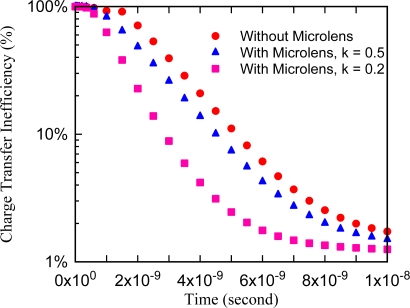
Electron transit time with on-chip microlens under 10,000e- illuminating condition.

Figure 8 shows the percentage of transferred electrons *vs.* time elapsing. The results are summarized as follows:

1. Without the microlens, 90% of electrons are transferred within 5 ns.
2. The microlens with a light collection rate of 50% is not so effective. However, if the collection rate is 20%, the efficiency is significantly improved and 97.2% of electrons reach the collection gate within 5 ns.

There are some further discussion topics on the electron transit time:

1. It is impossible to achieve transfer of 100% of electrons for 100 Mfps.
2. More than 90% can be transferred.
3. If we employ a microlens array and an appropriate post-processing technology, the frame rate very close to 100 Mfps can be achieved.

## 5. Technology to Deliver Driving Voltages

### 5.1. Metal Layout to Reduce RC Delay in Driving Voltage Transfer

As we move toward higher working frame rate, parasitic properties of wires, interconnections, and buried channel memories cause voltage drops which degrade the chip performance.

Typically, full chip circuit simulation by SPICE is time-consuming for preliminary layout design stage. Thus, it is important to develop a fast and sufficiently accurate expression that can be used to evaluate circuit performance of the chip.

Dao *et al*. [18] presented an efficient and accurate method for fast estimation of attenuation as well as delay of driving input pulses over a representative circuit of the chip. A first-order analytical model, *i.e.*, Elmore model, was used as the preliminary model. Later on, dimensional analysis was applied to carefully planned simulation cases to improve the accuracy of the preliminary model. The resultant expression is simple, closed-form with small estimation error. The method was used extensively for metal layout optimization of the ISIS-100M.

Our first attempt is based on the current foundry technology, which is a CCD process with two polysilicon layers and two metal layers. The top layer, aluminum is used as shunting wires within the imaging area as well as surrounding global bus lines. The lower layer, tungsten, is used mainly for bridging between the aluminum wires.

**Figure 9.**
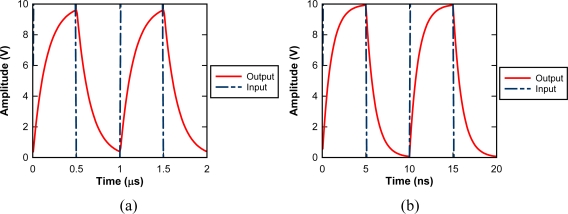
Input and output pair of A_1_ electrode: (a) With two-metal layer at 1 MHz (b) With three-metal layer at 100 MHz.

Figure 9 (a) shows circuit simulation results for one of a couple of electrodes to drive the two-phase transfer CCD, named as A1 and A2 electrodes. The attenuation of the driving pulse is large, even for 1 Mfps operation.

To increase the frame rate, we introduced the following two technologies:

1. An additional aluminum layer used as bus lines placed on the imaging area.
2. Direct bonding of wires from external circuitry to each bus line.

Originally, ten internal aluminum wires are placed on a pixel to drive various gate electrodes and to define voltages of the n and p substrates. However, during an image capturing phase, only the circuitry to store image signals operates and that for readout is fixed. The gate electrodes which operate for the image capturing and storing are only four electrodes, *i.e.*, the A1 and A2 electrodes for the storage CCD, the collection gate CL and the input gate IN, which is the first CCD element of the storage and transfers signal electrons from CL to the CCD storage. Therefore, the additional second aluminum layer is exclusively allocated to the metal wires on the imaging area to deliver power to these four electrodes, named the internal bus lines. Among them, the capacitive loads of the A1 and A2 electrodes covering most of the pixel area are dominant, to which the widest internal bus lines are allocated.

**Figure 10.**
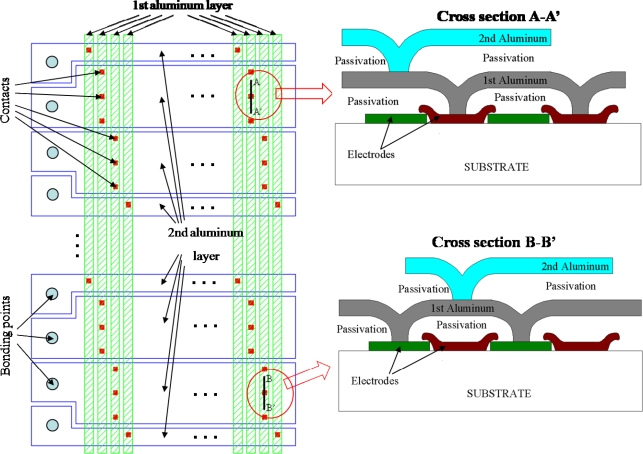
Bus line arrangement of the ISIS-100M.

The layout of the internal bus lines is conceptually depicted in Figure 10. On one quadrant of the sensor, eleven sets of the internal bus lines for A1, A2, CL and IN electrodes are placed. Each set covers 20 rows of pixels: widths of A1/A2, and CL/IN bus lines are respectively 400 and 90 micrometers; the thickness is 0.8 micrometers.

Attenuations of input driving voltages for the A1 and A2 internal bus lines at 100 Mfps are shown in Figure 9 (b). Attenuations of input driving voltages for the CL and IN bus lines are much smaller than those of A1 and A2 bus lines due to much lower parasitic capacitances. Thus, this design increased the driving power more than 100 times, and making image capturing at 100 MHz possible.

A serious problem associated with this design is that eighty eight high-power IC drivers have to be placed on each side of the sensor. The authors are developing a dedicated power IC driver array with an element driver with a width of 0.5 mm and an integrated packaging method.

### 5.2. Application of Differential Bus Lines

Now, we will further modify the metal wiring layout to make reactance part much smaller than resistance part in the impedance, we have chosen a critical condition of ωL < 0.1R as a design target.

As operating frequency increases, wiring parasitic inductance becomes a major concern which causes additional delay and coupling noises as follows:

1. In order to reduce RC delay, the global bus lines have to be wide and thick enough to reduce their resistances. This makes reactance part comparable or even greater than resistance part in the parasitic impedance.
2. As the frequency increases, driving voltage waveform contains higher frequency components, which makes the inductance effect more significant.

**Figure 11.**
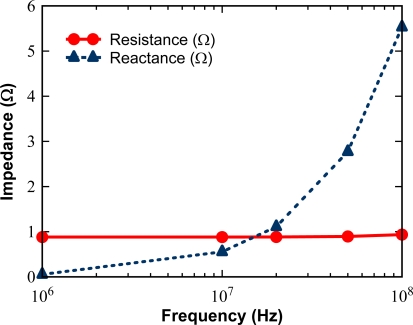
Impedance of A1/A2 bus lines *vs.* frequency.

Figure 11 shows comparison of resistance and reactance of an internal bus line for an A1/A2 electrode shown in Figure 10. The capacitive load to each A1/A2 internal bus lines is 2 nF, distributed uniformly in the longitudinal direction. A three-dimensional field solver, FastHenry, is used for the evaluation [19].

Figure 11 shows that:

1. Skin effect appears on resistance for frequencies larger than 20 MHz. However, up to 100 MHz, the effect can be neglected in practical designs.
2. Effect of magnetic inductance is very large. The condition *ωL* = 0.1R is obtained at a frame rate of 2 Mfps, much slower than our design target of 100 Mfps.

Therefore, the authors introduced a new design method specific to CCD and applied to the final design of the ISIS-100M.

It is well known that a two-phase CCD is driven by a couple of driving voltages with exact inverted patterns. A four phase CCD can be operated by two couples of inverted driving voltages. Then, directions and magnitudes of resulting currents on the coupled wires are always anti-phased and exactly the same.

It is also well known that differential signaling efficiently reduces the magnetic field. The currents flowing in opposite directions with the same magnitude compensate the magnetic fields generated by each wire. Traditionally, the concept has been utilized in twisted wires.

We applied the differential transmission to the internal bus lines for the A1/A2 electrodes. The resultant magnetic energy fields are dramatically decreased as shown case (c) of Table 3, which satisfies the condition: ωL < 0.1R.

Conventionally, in IC design, the differential transmission is commonly formed by sending the same signals from the both sides of a chip. For CCDs, since a pair of driving voltages is originally exactly anti-phased, the pairs can be sent from one side to the center, which makes the length of the wires a half of the chip width. Then, both the impedance and the capacitive load are halved; the delay or attenuation of the driving voltages decreases to a quarter; and, thus, the maximum frame rate subject to the phase-shift and/or the attenuation of the driving voltages are multiplied to four times.

### 5.3. Crossed Bundling

One problem associated with the overlapping is that upper bus line has no contact with the polysilicon electrodes to directly operate the CCD beneath the couple of bus lines.

**Figure 12.**
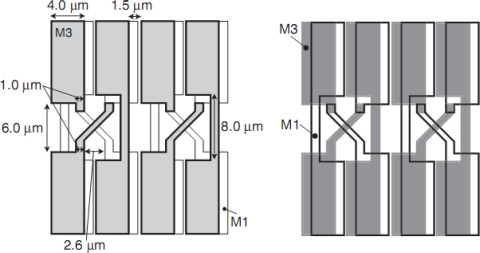
Twisted differential transmission line structure by Ito *et al*. [20]

**Figure 13.**
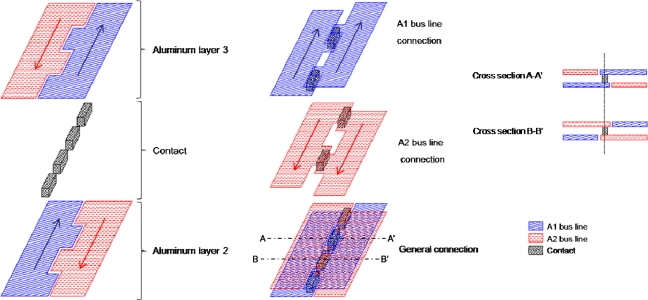
Crossed bundling concept for A1 and A2 bus lines.

Ito *et al*. proposed a twisted differential transmission lines with four metal wires [20] as shown in Figure 12. The stacked wires are twisted in the stretching direction and, thus, in the direction of the currents. The authors proposed a crossed differential transmission lines, shown in Figure 13. The wires are electrically connected in the direction perpendicular to the stretching direction through contact elements at the concavoconvex edges. The currents through the contact elements of this configuration are much smaller than those of the twisted one.

Standard CCD processes uses two or three polysilicon layers and two or three metal layers. The crossed differential transmission bus lines require additional one or two more metal layers. An alternative method is to place stacked wires on the carrier substrate on the ISIS-100M. Conventional backside illuminated image sensors are flipped and pasted on a carrier substrate. The voltages and signals are transferred through metal wires on the carrier substrate. The ISIS-100M is planned to be fabricated by a standard CCD process with three aluminum layer. The top aluminum layer contacts with the upper aluminum layer of the crossed differential bus lines on the carrier substrate. This configuration practically eliminates the inductance effects. Then, it is possible to achieve 100 Mfps. The test sensor is under fabrication.

## 6. CCD-CMOS Hybrid Ultra-High-Speed Image Sensor: RA-ISIS

The CMOS processes utilize four or more metal layers. Therefore, it is straightforward to design and fabricate the CCD-ISIS with the advanced CMOS process. The following technological advancements have made possible the CCD fabrication by CMOS processes:

1. With a gap narrower than 0.2 μm between adjacent transfer gate electrodes, a smoothly changing potential profile is created in the buried CCD channel. Recent deep-submicron CMOS processes allow fabrication of polysilicon gates with the gap less than 0.2 μm.
2. Only minor process modification is required. To increase full well capacity and create a smooth channel potential beneath the poly-poly gap, thickening of the oxide insulation layer is necessary.
3. Various other CCD related technologies had been introduced to the advanced CMOS image sensor process such as deep implanting, floating diffusion amplifier, correlated double sampling technique, *etc*.

CCD fabrication using the CMOS imager process has been investigated thoroughly. Even an image sensor with 0.5-μm CCD channel pitch has been fabricated by using 0.11-μm CMOS process [15].

The CMOS-based CCD-ISIS is a promising way to fabricate the ultimate ultra-high-speed image sensor for the following reasons:

1. Advanced CMOS processes utilize four or more metal layers, from which two upper metal layers are allocated to the crossed differential internal bus lines to significantly reduce the inductance.
2. The very fine design rule makes possible scaling of the CCD-ISIS, which is the simplest way to increase the maximum frame rate. For example, when the driving voltage transfer is the limiting factor on the maximum frame rate, by reducing the width of the chip to a half of the original size, resistance and inductance of the internal bus lines and the capacitance of the buried CCD channels all reduces to the halves of the original values. As a result, the total power load for the driving voltage transfer is reduced to a quarter, which increases the maximum frame rate four times.
3. Incorporating CMOS readout technology with CCD *in situ* storage, we can obtain an ultimate high speed image sensor with flexible and high speed readout by CMOS technology and ultra-high-speed and superior image quality by CCD technology.

Therefore, the authors have changed the strategy to reach 100 Mfps. The ISIS-100M currently under fabrication will be used for evaluation of fundamental performances. Based on the evaluation, the authors plan to develop the RA-ISIS, capable of random-access readout by CMOS circuitry and ultra-high-speed image capturing by CCD *in situ* storage.

## 7. Other Technical Problems

There are some other problems and tasks to achieve 100 Mfps as follows:

1. Heat generation
2. Removal of positive charge carriers (holes) from the backside
3. Compact high-power driver IC
4. Integrated packaging

Continuous overwriting operation at an ultra-high image capturing operation generates and accumulates heat. For example, the camera mounting the ISIS-V2 [2,3] continuously operates only for 30 seconds for image capturing at 1 Mfps to avoid too much increase of the temperature of the sensor.

Incident photons generate electron-hole pairs. Holes are collected at the backside. For a very high frame rate, a metal stripe or grid is to be placed on the backside to effectively drain the large hole current. Development of the technology is one of the major issues for the next-stage development.

However, it is expected that capturing 126 image frames without overwriting operation generates moderate temperature rise at an instance and a limited number of holes. At the first development stage, we will test ISIS-100M by taking images of exactly 126 frames to avoid these problems.

There is no high-power and compact driving IC in the market capable to drive 2nF capacitive load distributed along each internal bus line at 100 MHz. Larger ICs cannot be implemented close to the image sensor, which significantly increases inductance along long wires delivering the power to the sensor. The compact high-power drivers have to be implemented on the package surface next to the image sensor or in the carrier substrate.

The importance of development of a high power driving IC and an integrated system was pointed out by scientists of the International Linear Collider (ILC) project [21,22]. They have developed an ASIC to drive 40nF at 50 MHz with a clock drive voltage of ±2 V Much higher clock drive voltage (up to ±8V) is needed to achieve high dynamic range images with the ISIS-100M. Therefore, in parallel to development of the ISIS-100M, the authors are developing a dedicated high-power, high voltage CMOS driver and an integrated packaging method of the image sensor.

## 8. Conclusions

The design experience of an image sensor to achieve 100 Mfps with very high sensitivity is presented. The frame rate is enabled by the CCD-ISIS structure; the sensitivity by backside illumination and cooling. The authors have developed an ultra-high sensitivity image sensor with the maximum frame rate of 1 Mfps. Various barriers against further increases in the frame rate are listed. Original technologies to overcome the difficulties are proposed and tested by simulations, as follows:

1. The time required for photo-electrons generated at the backside to reach the storage CCD on the front side is minimized by employment of the wafers with double-epi layers, the special three-layer p-well design, the curved CCD design and a microlens array.
2. The RC-delay is minimized by introducing one additional layer for the internal bus lines and directly bonding them to wires from external circuitry. A simple estimation method developed by the authors contributes to optimization of the metal wiring.
3. Inductance of the internal bus lines is minimized by the proposed crossed differential bus lines, which is specifically efficient to drive CCDs.
4. The chip size is minimized to fabricate the CCD-ISIS using an advanced sub-micron CMOS image sensor process.

The fusion of CMOS and CCD imager technologies enables realization of RA-ISIS, the random-access *in situ* image sensor, which is the ultimate high-speed and highly-functional image sensor. On a simulation basis, it is confirmed that the modified ISIS-100M operates at 100 Mfps with support of the proposed technologies. However, there is no driver IC in market to have sufficient power to drive the ISIS-100M and compact enough to be distributed closely around the sensor chip to reduce both resistive and reactive impedance. To achieve 100 Mfps, it is important to develop an integrated system with all relating parts together under a comprehensive plan.

## Figures and Tables

**Table 1.** Development of ISIS image sensors at Kinki University, Japan.

**Sensor/Camera**	**Frontside illuminated**		**Backside illuminated**	
	**ISIS-V2 [2,3]**	**ISIS-V4 [4]^+^**	**ISIS-V12 [5,13]**	ISIS-100M^*^
**Pixel count (pixels)**	81,120 (312 × 260)	302,400 (420 × 720)	201,600 (480 × 420)	140,800 (440 × 320)
**Maximum frame rate**	1 Mfps	1 Mfps	1 Mfps	100 Mfps
**Technologies for high sensitivity**	N/A	N/A	CCM/Backside illumination/Cooling	Backside illumination/Cooling

* indicates that the sensor is currently under fabrication.

+ indicates that the sensor is jointly developed with NHK, a Japanese public broadcasting company.

**Table 2.** Design specification of the ISIS-100M.

Frame Rate	30–100,000,000 fps
Pixel Count	440 × 320 (=140,800) pixels
Pixel Size	50.4 × 50.4 μm^2^
CCD element size	3.6 × 3.6 μm^2^
Number of Stored Images	126 frames
Full well Capacity	8,000 electrons
Grey Level	>9 bits
Overwriting Drain	Installed
On-chip trigger system	Installed
Transfer Scheme	2-phase transfer for VCCD, voltage swing 10 V 4-phase transfer for HCCD, voltage swing 5 V

**Table 3.** Implementations for on-chip inductance reduction. Wire thickness, width and length are 0.8; 400 and 10,000 μm respectively.

Cases	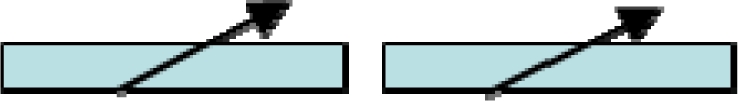 (a)	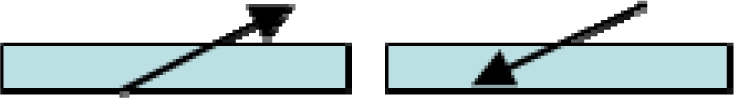 (b)	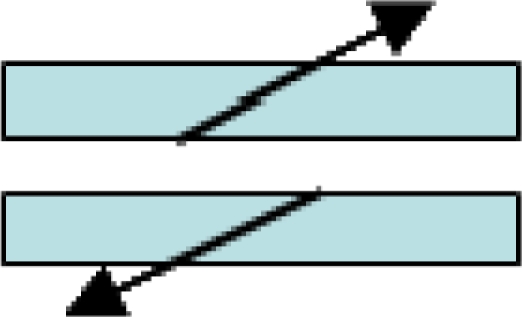 (c)
Magnetic energy (ME) profile	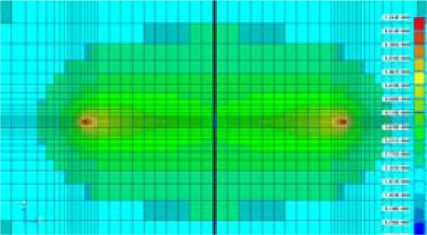	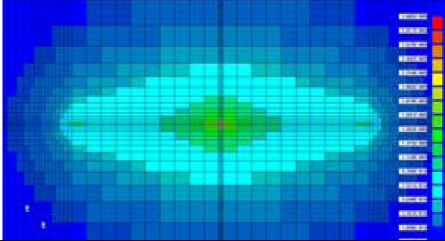	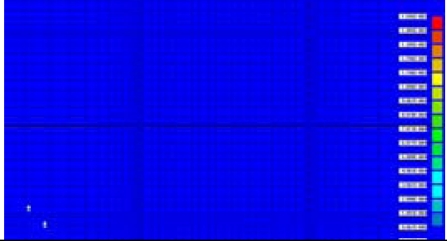
Relative ME	100%	25%	**0.5%**
ωL/R	600%	150%	**3%**
